# Retraction: Leptin Induces Cyclin D1 Expression and Proliferation of Human Nucleus Pulposus Cells via JAK/STAT, PI3K/Akt and MEK/ERK Pathways

**DOI:** 10.1371/journal.pone.0282978

**Published:** 2023-03-29

**Authors:** The PLOS ONE Editors

The *PLOS ONE* Editors retract this article [1] because it was identified as one of a series of submissions for which we have concerns about peer review, authorship, and similarities across articles. Image similarities were noted between Fig 1D in [1] and Fig 4A in [2], as well as between Fig 3B and 7B in [1] and Fig 2C in [3]. These concerns call into question the validity and provenance of the reported results. We regret that the issues were not addressed prior to the article’s publication.

GQ and JS agreed with the retraction. ZL did not agree with the retraction. XY, JLiang, and JLiu either did not respond directly or could not be reached. WKKW responded but expressed neither agreement nor disagreement with the editorial decision.
